# Decompressive Craniectomy: From Ancient Practices to Modern Neurosurgery

**DOI:** 10.7759/cureus.64923

**Published:** 2024-07-19

**Authors:** Chahat Singh, Pankaj Gharde, Sandeep Iratwar, Prince Verma, Bhushan Triwedi

**Affiliations:** 1 General Surgery, Jawaharlal Nehru Medical College, Datta Meghe Institute of Higher Education and Research, Wardha, IND; 2 Neurosurgery, Jawaharlal Nehru Medical College, Datta Meghe Institute of Higher Education and Research, Wardha, IND

**Keywords:** ethical considerations in surgery, pediatric neurosurgery, traumatic brain injury (tbi). stroke malignant edema, intracranial pressure (icp), decompressive craniectomy

## Abstract

Decompressive craniectomy (DC) is a neurosurgical strategy that expels a parcel of the cranium to relieve pressure on a swollen or herniating brain. This review article explores the history of DC, from its ancient roots in trepanning to its contemporary applications. It then examines the mechanisms by which DC reduces intracranial pressure (ICP) and improves cerebral blood flow. The article highlights the efficacy of DC in treating patients with severe traumatic brain injury (TBI), stroke, and other conditions that cause increased ICP. However, it also acknowledges the potential complications of DC, such as infection and bleeding. The ethical considerations surrounding DC are explored in detail, particularly the challenging decision-making process for patients who are unable to give consent. A specific focus is given to the use of DC in pediatric patients, where the developing brain is especially vulnerable to pressure changes.

## Introduction and background

Decompressive craniectomy (DC) is a surgery in which a portion of the skull is removed to provide space for a herniating brain to expand without being compressed [1,2]. Patients with traumatic brain injury (TBI), stroke, and other disorders linked to elevated intracranial pressure are treated with DC [3,4]. Even though this trepanning procedure was popular in prehistoric times, it was phased out as a modern, less invasive treatment technique emerged [5,6]. Although it was still used before the twentieth century, its modern version was made possible by the invention of sophisticated post-operative care such as antibiotics, cranial drills, and precision cutting tools [6,7].

## Review

Search methodology

**Figure 1:**
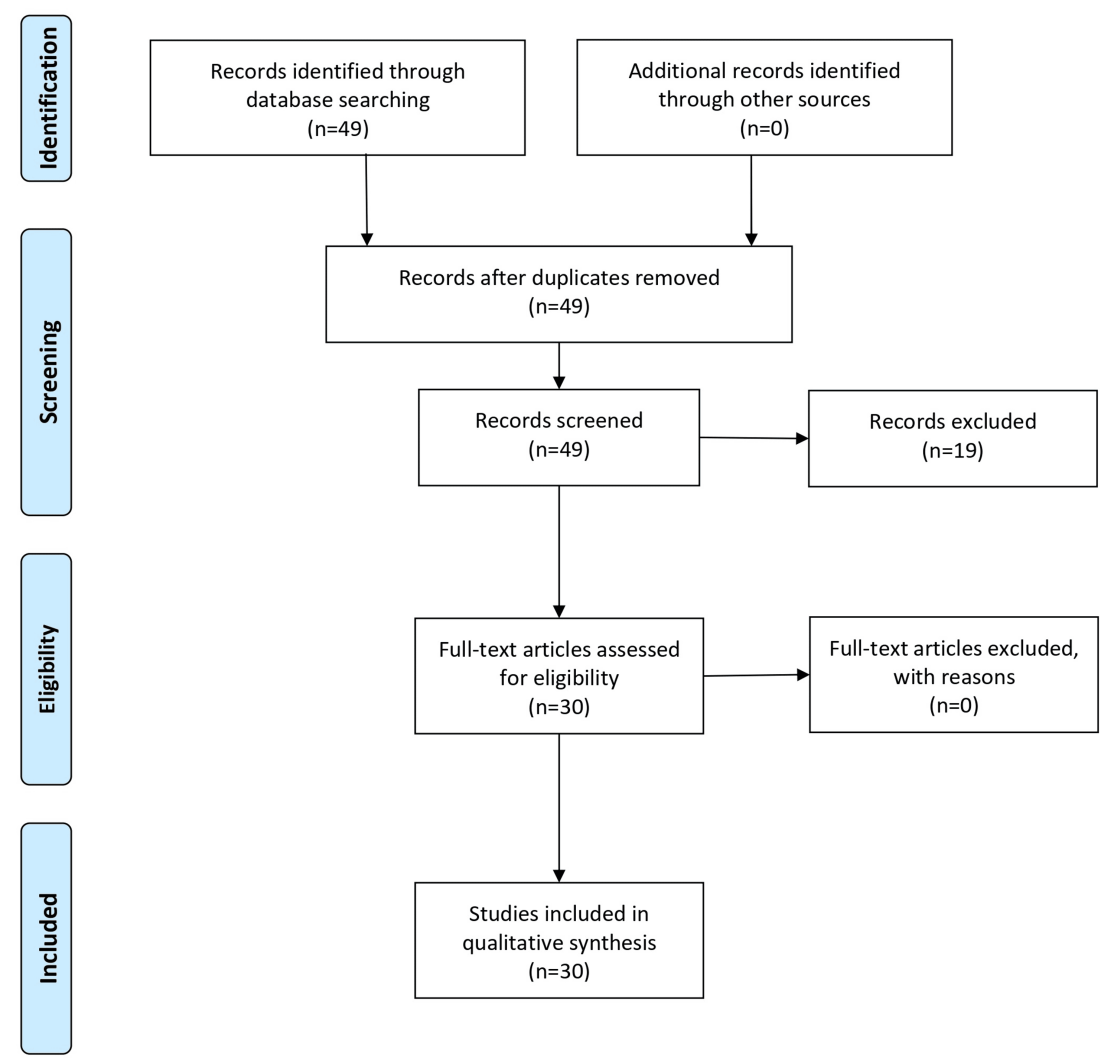
PRISMA flow chart. PRISMA: Preferred Reporting Items for Systematic Reviews and Meta-Analyses.

To form this review article on DC, a thorough search methodology was utilized. This included scouring databases like PubMed Central and MEDLINE for important articles. Search terms centered on DC, intracranial pressure (ICP), and its applications in treating brain wounds and strokes. Filters used in the search guaranteed articles were recent, i.e., within the last 10-15 years and in English. Ultimately, the search expanded beyond articles to significant websites and course readings to capture the most up-to-date information on DC. The Preferred Reporting Items for Systematic Reviews and Meta-Analyse (PRISMA) flow chart is shown in Figure 1.

Impact of DC on ICP and cerebral blood flow

Although DC is only used as a last option, some research indicates that it can enhance outcomes by reducing ICP or the pressure within the skull [8,9,10]. Due to the compression of the brain and the restriction of cerebral blood flow, elevated ICP frequently results in fatalities or severe disabilities [8]. It aims to lower this pressure that DC is utilized. The section of the skull that is excised is called a bone flap [11,12]. ICP is lowered more when a larger bone flap is removed [12].

The outcomes of the Decompressive Craniectomy (DECRA) trial were released by Australian and international scientists in The New England Journal of Medicine in March 2011 [13]. This was a randomized experiment conducted between 2002 and 2010 to determine the best course of action for patients with diffuse non-penetrating head injuries whose ICP was medically resistant [13]. The trial compared DC to best medical care. Many neurosurgeons in practice have rejected or at least questioned the DECRA trial results, and an editorial published at the same time points out various flaws in the study [13]. Research has shown that DC improves cerebral blood flow and cerebral perfusion pressure in individuals with head injuries in addition to lowering ICP [14,15].

DC for Malignant Edema and ICP in Stroke Patients

Major strokes linked to 'malignant' edema and ICP are also treated with DC [16,17]. The pooled data from three randomized controlled trials conducted in Europe confirms the retrospective findings that, when compared to conservative management alone, early (within 48 hours) application of DC following 'malignant' stroke may improve survival and functional outcome in patients under the age of 55 [18]. Particularly for young children whose ICP cannot be controlled by other means, DC is advised [10,19]. A worse outcome following DC is linked to age higher than 50 [20].

Ethical dilemmas in deciding to perform DC

DC is a surgical method that raises a number of ethical issues, chief among them the concepts of beneficence and non-maleficence [4]. Medical professionals must act in the patient's best interest to be considered beneficial; in cases of severe TBI or stroke, this frequently implies promoting DC as a life-saving treatment [7,19]. However, the concept of non-maleficence, which requires individuals to refrain from harming others, balances this out. Noteworthy risks associated with the operation itself include bleeding, infection, and the potential for further brain damage. Moreover, the outcomes are uncertain; DC does not guarantee an effective recovery, even though it can lower ICP and save impending death. Since patients may survive with significant neurological damage, medical professionals must carefully consider whether the procedure's short-term advantages outweigh any potential long-term risks [13].

The moral scene gets much more complicated when a patient is unfit of giving educated authorization or is disabled. Family individuals or legitimate operators are then left in charge of making decisions on the patient's behalf. Due to the vulnerability encompassing the patient's prognosis and their fear of making the incorrect decision, these surrogates habitually experience extreme emotional and moral strain [14]. There are noteworthy concerns over the patient's future independence and quality of life if they are to survive for an extended period of time while severely disabled. Healthcare experts must handle these delicate discussions with care, offering the patient and their loved ones compassionate, clear advice while honoring their beliefs and preferences [7,11]. The decision to conduct DC is one of the most difficult ethical dilemmas in critical care because it highlights the moral conflict between ensuring life at all costs and honoring the patient's autonomy, dignity, and capacity for suffering [14]. Ethical considerations in DC are described in Table 1.

**Table 1:** Ethical considerations in decompressive craniectomy. Table created by Singh C. Reference: [4, 7, 11, 13, 14] DC: Decompressive craniectomy.

Concept	Description	Reference
Beneficence vs. non-maleficence	Medical professionals must prioritize the patient's well-being (beneficence). DC can be life-saving in severe brain injuries.	[4]
Uncertain outcomes	DC reduces pressure but doesn't guarantee recovery. Patients might survive with significant neurological impairments.	[13]
Decision-making for incapacitated patients	When patients are unable to provide consent, families or guardians make the decision.	[14]
Quality of life concerns	If patients survive with severe disabilities, their future independence and quality of life become a concern.	[7,11]
Conclusion	The decision to perform DC is a major ethical dilemma in critical care. It involves balancing life preservation with patient autonomy, dignity, and potential suffering.	[14]

Outcomes of DC in pediatric patients

According to some research, all children who underwent DC for serious head injuries recovered well, indicating that the surgery may be superior to non-surgical care for children with similar injuries [21,22]. When tracked for more than five years, pediatric patients with unintentional trauma following a craniectomy had a net 65% good outcomes rate, according to one of the largest studies on pediatric patients, conducted by Jagannathan et al [23]. Due to the distinct anatomical and physiological traits of children's brains, which are more sensitive to variations in pressure and volume, DC is especially important in pediatric patients [16,24]. DC may boost overall neurological outcomes, lower the likelihood of brain herniation, and improve cerebral perfusion [25]. Given the continuous growth and development of the skull and brain in pediatric patients undergoing DC, particular attention is paid to the size and position of the bone flap [26]. When deciding whether to perform DC, one must consider both the short- and long-term effects, particularly for developing children [24].

Brain damage is more likely to occur following a craniectomy, especially as the patient recovers and regains movement [8,27]. As a result, further precautions need to be taken to safeguard the brain, such as wearing a helmet or placing a temporary implant in the skull [15,28]. A cranioplasty is often used to seal the skull hole once the patient has healed adequately [29]. After the craniectomy, if at all feasible, the original part of the skull is kept reserved for the cranioplasty procedure [30]. DC in children with head injuries is described in Table 2.

**Table 2:** Decompressive craniectomy in children with head injuries. Table created by Singh C. Reference: [16, 23, 24, 25, 26] DC: Decompressive craniectomy.

Feature	Benefit	Reference
Recovery rates	High rates of good outcomes (65%) for unintentional trauma after craniectomy (>5 years)	[23]
Importance in pediatrics	More sensitive brains due to ongoing development	[16, 24]
Potential benefits of DC	Improved neurological outcomes, reduced brain herniation risk, and better cerebral perfusion	[25]
Considerations for DC	Size and placement of bone flap due to skull/brain growth	[26]
Short and long-term effects	Weigh both benefits and risks for developing children	[24]

DC mechanism in reducing ICP

By excising a portion of the skull, DC decreases ICP and permits unfettered growth of the enlarged brain [11]. When a patient has a severe TBI, stroke, or any illness that causes significant brain edema and high ICP, this intervention is critical. The removal of a part of the skull helps to provide room for the growing brain by enlarging the hard boundaries of the cranial cavity [15]. This drop in pressure can lessen the risk of brain hernia, a condition in which the brain is pushed through internal cranial structures, by preserving cerebral circulation and preventing further brain tissue damage [1,13].

The degree to which ICP is reduced during DC is significantly influenced by the location and size of the bone flap as well as the amount of the skull that is removed [9,11]. A larger bone flap is often associated with a greater reduction in pressure because it provides more space for the developing brain to expand [15]. When surgery is performed on one side, the bone flap usually covers the temporoparietal area; however, when edema is more extensively distributed, the bilateral frontal regions are usually covered [17]. Based on imaging examinations and the specific site of damage or edema, the precise region and size are identified. Table 3 shows a DC for reducing ICP.

**Table 3:** Decompressive craniectomy for lowering intracranial pressure. Table created by Singh C. Reference: [1, 9, 11, 13, 15, 17]

Feature	Description	Reference
Purpose	Lower intracranial pressure (ICP)	[11]
Indication	Severe traumatic brain injury, stroke, or infection causing brain edema and elevated ICP	[11]
Mechanism	Creates space for swollen brain tissue by removing a portion of the skull	[15]
Benefit	Reduces risk of brain herniation, preserves cerebral circulation, prevents further brain damage	[1,13]
Key factor for ICP reduction	Size and placement of bone flap (removed skull section)	[9,11]
Larger bone flap effect	Greater ICP decrease due to increased space for brain expansion	[15]
Bone flap location	Unilateral surgery: temporoparietal region (most swollen area) - Bilateral edema: bilateral frontal areas	[17]
Determination of location and size	Imaging studies and specific injury/edema location	[17]

## Conclusions

DC is a critical surgical method utilized to treat life-threatening conditions that cause increased ICP and swelling within the brain. It offers a potential life-saving option for patients with severe TBI, stroke, and other neurological emergencies. DC works by removing a portion of the cranium to create space for the brain to expand, thereby reducing pressure and preventing further damage. Research suggests that DC can effectively lower ICP and improve cerebral blood flow. However, the decision to perform DC is complex and requires consideration of both the potential benefits and risks. While DC can increase survival rates, it does not guarantee full recovery. Patients may experience significant neurological disabilities and require long-term restoration.

DC carries some surgical risks, such as bleeding, infection, and further brain injury. The ethical considerations surrounding DC are particularly challenging. Medical professionals must weigh the principles of beneficence (doing good) and non-maleficence (avoiding harm) when deciding whether to proceed with surgery. Families of patients who are incapable of giving their consent for surgery may be left to make difficult decisions, which can be emotionally and ethically burdensome. Because of the extreme sensitivity of a child's developing brain, DC can be particularly important for pediatric patients with severe head injuries. In any case, to account for ongoing skull and brain development in children, the precise size and location of the bone flap removed during DC must be given careful consideration. DC is still, overall, a successful method for treating severe neurological disorders. It is a complicated and nuanced process that calls for careful assessment of the benefits and risks involved as well as a thorough understanding of the ethical issues raised.
